# Immediate Sample Fixation Increases Circulating Tumour Cell (CTC) Capture and Preserves Phenotype in Head and Neck Squamous Cell Carcinoma: Towards a Standardised Approach to Microfluidic CTC Biomarker Discovery

**DOI:** 10.3390/cancers13215519

**Published:** 2021-11-03

**Authors:** Karl Payne, Jill M. Brooks, Graham S. Taylor, Nikolaos Batis, Boris Noyvert, Yi Pan, Paul Nankivell, Hisham Mehanna

**Affiliations:** 1 Institute of Head and Neck Studies and Education, Institute of Cancer and Genomic Sciences, University of Birmingham, Birmingham B15 2TT, UK; j.m.brooks@bham.ac.uk (J.M.B.); n.batis@bham.ac.uk (N.B.); p.c.nankivell@bham.ac.uk (P.N.); h.mehanna@bham.ac.uk (H.M.); 2 Institute of Immunology and Immunotherapy, University of Birmingham, Birmingham B15 2TT, UK; g.s.taylor@bham.ac.uk; 3 Cancer Research UK Birmingham Centre, University of Birmingham, Birmingham B15 2TT, UK; b.noyvert@bham.ac.uk (B.N.); y.pan@bham.ac.uk (Y.P.); 4 Centre for Computational Biology, University of Birmingham, Birmingham B15 2TT, UK

**Keywords:** circulating tumour cell, head and neck cancer, head and neck squamous cell carcinoma, microfluidic enrichment, Parsortix, Transfix

## Abstract

**Simple Summary:**

Circulating tumour cells (CTCs) have shown potential to act as markers of disease and prognosis in head and neck squamous cell carcinoma (HNSCC). However, there are a number of methods and devices reported to isolate and characterise CTCs. Translating CTC markers to clinical practice, for patient benefit, requires a reliable, reproducible and standardised approach. We report the benefit of the Parsortix microfluidic CTC enrichment platform in HNSCC. We demonstrate consistent cell capture rates between 10 and 100 cells/mL of whole blood. Analysis of gene expression with unfixed cells before and after Parsortix enrichment demonstrated a cell stress response and downregulation of key genes. We highlight the benefit of using a fixative blood collection tube (Transfix) to increase cell capture rate and preserve the CTC marker expression profile. Such evidence is crucial when designing sample processing protocols for large cohort multi-centre clinical trials investigating CTCs in any cancer type.

**Abstract:**

Introduction: Research demonstrates strong evidence that circulating tumour cells (CTCs) can provide diagnostic and/or prognostic biomarkers in head and neck squamous cell carcinoma (HNSCC) and a potential tool for therapeutic stratification. However, the question still remains as to the optimum method of CTC enrichment and how this can be translated into clinical practice. We aimed to evaluate the Parsortix microfluidic device for CTC enrichment and characterisation in HNSCC, seeking to optimise a sample collection and processing protocol that preserves CTC integrity and phenotype. Method: Spiking experiments of the FaDu and SCC040 HNSCC cell lines were used to determine the Parsortix capture rate of rare “CTC-like” cells. Capture rates of cancer cells spiked into EDTA blood collections tubes (BCTs) were compared to the Transfix fixative BCT and Cytodelics whole blood freezing protocol. The Lexogen Quantseq library preparation was used to profile gene expression of unfixed cells before and after microfluidic enrichment and enriched cell line spiked Transfix blood samples. An antibody panel was optimised to enable immunofluorescence microscopy CTC detection in HNSCC patient Transfix blood samples, using epithelial (EpCAM) and mesenchymal (N-cadherin) CTC markers. Results: Across a spiked cell concentration range of 9–129 cells/mL, Parsortix demonstrated a mean cell capture rate of 53.5% for unfixed cells, with no significant relationship between spiked cell concentration and capture rate. Samples preserved in Transfix BCTs demonstrated significantly increased capture rates at 0 h (time to processing) compared to EDTA BCTs (65.3% vs. 51.0%). Capture rates in Transfix BCTs were maintained at 24 h and 72 h timepoints, but dropped significantly in EDTA BCTs. Gene expression profiling revealed that microfluidic enrichment of unfixed cell lines caused downregulation of RNA processing/binding gene pathways and upregulation of genes involved in cell injury, apoptosis and oxidative stress. RNA was successfully extracted and sequenced from Transfix preserved cells enriched using Parsortix, demonstrating epithelial specific transcripts from spiked cells. In a proof-of-concept cohort of four patients with advanced HNSCC, CTCs were successfully identified and visualised with epithelial and epithelial-mesenchymal phenotypes. Conclusion: We have optimised a protocol for detection of CTCs in HNSCC with the Parsortix microfluidic device, using Transfix BCTs. We report a significant benefit, both in terms of cell capture rates and preserving cell phenotype, for using a fixative BCT- particularly if samples are stored before processing. In the design of large cohort multi-site clinical trials, such data are of paramount importance.

## 1. Introduction

The ability to utilise a liquid biopsy to provide prognostic or predictive biomarkers is a marker of significant advances in multiple cancer types [1]^.^ A liquid biopsy is a blood test that, in comparison to a solid tissue biopsy, is less morbid and increasingly cost-effective, with the capability of performing serial measurements [2]. This is particularly beneficial as a tool for post-treatment surveillance. Circulating tumour cells (CTCs) are one compartment of a liquid biopsy that have generated considerable interest, promising the ability to provide tumour-specific proteogenomic data at the cellular level, which is not seen in circulating tumour DNA [3]. In the post-treatment setting, this offers the potential to provide biomarkers for disease surveillance and guide targeted therapy in patients with recurrent/metastatic disease. In addition, CTCs allow a “real-time” picture of temporal tumour genomic/molecular heterogeneity not afforded by spatially biased tissue biopsies [4]. With high rates of recurrence/metastasis (R/M) and 5-year survival that has largely plateaued at 50–60% [5], the treatment of head and neck squamous cell carcinoma (HNSCC), particularly in the R/M setting, remains challenging. In particular, tumour sites are often not amenable to repeat biopsies and thus clinicians are blind to evolving temporal heterogeneity. While the advent of immune-modifying treatment modalities offers a new therapeutic approach, significant improvements in outcome are only likely to be realised with the ability to make biomarker-led treatment decisions for these targeted therapies. Utilising CTCs as a source of predictive and/or therapeutic biomarkers to improve patient stratification in these cohorts holds particular promise [1].

Strategies to isolate CTCs are broadly classified as either marker-dependent (utilising cell surface antigens) or marker-independent (assessing cell biophysical properties) [6]. Those methods that used epithelial markers for positive selection gained early interest, for example the EpCAM-dependent CellSearch^®^ platform. However, several reports have identified additional markers that can be used to characterise CTC sub-populations, for example mesenchymal or stemness markers [7], in addition to expression of druggable targets such as EGFR [8,9] or PD-L1 [10]. Therefore, the application of microfluidic technology to enrich cells based upon biophysical properties has rapidly become an area of interest, opposing the epithelial marker bias of previous positive selection platforms [6]. Early research in HNSCC sought to establish CTC presence or count as a diagnostic tool or prognostic biomarker for survival outcomes [11]. More recently, research has focused on multiparameter characterisation, seeking to further define CTC phenotypic markers and how they relate to treatment outcomes or have the potential to define therapeutic biomarkers, such as PD-L1 expression [7,8,9,10,12]. Thus, the ideal platform should provide a reproducible protocol for marker-independent CTC isolation.

As CTC research progresses towards clinical utilisation, there is a need to standardise sample collection and processing. Currently, no universally accepted method exists for CTC isolation/enrichment or sample collection and time to processing, making it difficult to directly compare results between studies. The methods of sample collection and pre-processing protocols (i.e., storage time) are often overlooked or underreported in studies, despite evidence of the significant impact these variables can have upon CTC capture and phenotype characterisation [13,14,15].

**Figure 1:**
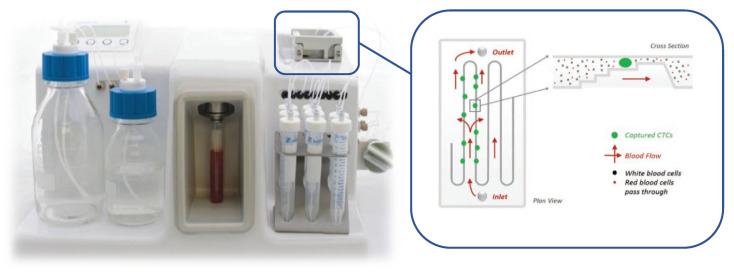
The Parsortix device (blood tube loaded in place) with diagrammatic representation of microfluidic flow and cell sorting within the isolation cassette, demonstrating tiered multi-channel design (pictures reproduced with permission of Angle Plc).

Our primary aim was to establish a protocol that enabled samples to be easily collected and stored for several days to allow a timeframe for transport from multiple sites, as expected in future large cohort clinical trials. We chose to evaluate the Parsortix platform in HNSCC CTC analysis because it provides a standardised approach to CTC enrichment with inbuilt protocols for various applications and has shown success in several other cancers [16,17]. The Parsortix instrument utilises a microfluidic cassette with a “tiered” internal structure to separate cells based on size and deformability characteristics (Figure 1). We sought to validate this platform in a HNSCC model to test the feasibility of using a CTC preservative blood collection tube (BCT) to stabilise blood samples prior to Parsortix enrichment, testing the reliability of enumeration and transcriptomic characterisation of enriched CTCs.

## 2. Materials and Methods

### 2.1. Cell Lines and Cell Culture

The SCC040 (UPCI-SCC-040; ACC-660; DSMZ, Brunswick, Germany), FaDu (ATCC HTB-43) and CAL27 (ACC-446; DSMZ) HNSCC cell lines were used for spiking experiments and as positive controls for immunofluorescent staining. Cell lines were cultured as previously described [18].

### 2.2. Patient and Healthy Donor Samples

HNSCC patients were recruited via the AcceleraTED2 study (REC reference: 16/NW/0265). Patient (pre-treatment) samples were collected into 9 mL Transfix^®^ (Cytomark, Buckingham, UK) tubes, stored at 4 °C and processed within 24–48 h. Healthy donor (HD) blood was collected into EDTA (Ethylenediaminetetraacetic acid), Transfix or Cytodelics blood tubes as specified. Cytodelics-preserved blood samples were stored at −80 °C for a minimum of 24 h before processing.

### 2.3. Sample Collection and Processing

We investigated the impact of blood collection tube (BCT) and sample time to processing upon cell capture rate. Our rationale was to investigate whether a preservative (fixative)-based BCT impacted cell capture rate and if capture rate altered over time with differing BCTs-given that clinical samples are often not processed immediately, especially samples from multi-site trials. The Transfix BCT was compared to a standard EDTA BCT at 0 h, 24 h and 72 h storage timepoints, and both compared to the Cytodelics whole blood preservation kit. The Cytodelics kit (Stockholm, Sweden) was tested because it allows whole blood to be frozen (unprocessed) and stored for up to 1 year at −80 °C, thus raising the possibility of long-term storage of CTC samples, a strategy previously untested. Cytodelics-processed samples were frozen at −80 °C for 24 h before thawing and processing.

### 2.4. CTC Enrichment

CTCs or spiked HNSCC cells were enriched using Parsortix^®^ Technology (Angle plc, Surrey, UK) protocol *PX2_S99F* and a 6.5 μm separation cassette. For cell spiking experiments, HNSCC cells were labelled with CellTracker^TM^ Red (Invitrogen, Waltham, MA, USA) and added into HD blood at a target concentration of 10, 50 or 100 cells/mL. This concentration range was chosen because it is representative of reported CTC counts in HNSCC [7,8,19,20,21] and, importantly, it is comparable to concentrations used in other cancer type cell line spiking experiments with the Parsortix device [16,17], thus facilitating direct comparison. The actual cell number was confirmed by manual counting of labelled cells within the Parsortix enrichment cassette. Percentage capture rate was compared between samples in different blood collection tubes using a paired *t*-test.

The accepted definition of a CTC in HNSCC is an epithelial marker positive, leukocyte marker (CD45) negative, nucleated (DAPI+) circulating cell [22]. An antibody panel meeting these criteria was designed and optimised for “in-cassette” staining using protocol *PX2_stain1* (Table 1). The mesenchymal marker (N-cadherin) was included to identify CTCs undergoing an epithelial–mesenchymal transition (EMT). The enriched cell fraction was harvested from the cassette and slide mounted for immunofluorescence microscopy. This can be compared with previously published negative depletion protocols [7]; in some experiments, spiked HD blood was fractionated using lymphoprep density gradient centrifugation and CD45-positive peripheral blood mononuclear cells (PBMCs) were then depleted via magnetic-activated cell sorting (MACS; Miltenyi Biotech, Cologne, Germany).

### 2.5. RNA Sequencing

Extraction of RNA from unfixed samples was performed using the Qiagen (Hilden, Germany) RNEasy Micro kit. RNA from Transfix-preserved samples was extracted using the ThermoFisher Pico Pure kit. Library preparation was performed using the Lexogen’s Quantseq 3′mRNA-Seq FWD Library Prep method and sequencing performed on an Illumina NextSeq 500 platform. Sequencing reads were trimmed to remove adapter contamination, polyA read through, and low-quality tails using bbduk utility according to Lexogen guidelines. The trimmed reads were mapped to the GRCh38 (hg38) human genome with STAR aligner (v2.5.2b) [23]. Reads mapping to genes were counted by the same software. Normalisation of read counts and differential expression analysis was performed with the DESeq2 R Bioconductor package [24], taking into account the paired sample design of the experiment. Gene set enrichment analysis was performed using the GAGE (v.2.36.0) R Bioconductor package [25] with the Gene Ontology database.

## 3. Results

### 3.1. Parsortix CTC Enrichment in HNSCC Cell Line Model

**Figure 2:**
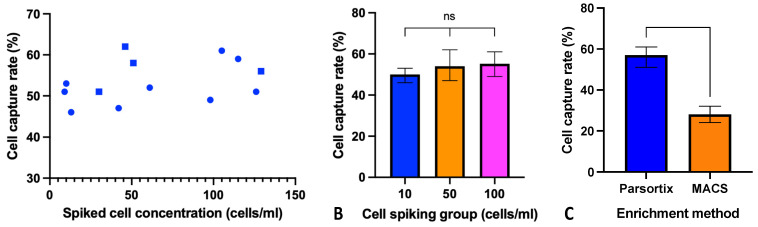
Spiked cell concentration does not impact capture rate, and microfluidic enrichment is superior to negative depletion protocol. (**A**) FaDu and SCC040 cells were spiked into 4 mL of healthy donor (HD) blood at the stated concentrations. Each symbol represents an individual experiment (FaDu cells round dots and SCC040 cells square dots). (**B**) Data set of capture rates in figure A grouped into target concentrations of ≈10, 50 and or 100 cells/mL. No significant difference between groups (*p* = 0.415). (**C**) FaDu cells were spiked into 4 mL of HD blood at concentrations of 100 cells/mL and enriched using Parsortix or CD45 depletion (MACS) to test cell capture rate (*n* = 3 per protocol). Comparison between Parsortix and MACS depletion was not performed on fixed cells, due to the incompatibility of lymphoprep density gradient centrifugation with fixed cells.

The initial work sought to verify the ability of Parsortix to capture HNSCC “CTC-like” cells using a cell line model, in which HD blood was spiked with FaDu or SSC040 HNSCC cells at a target concentration of 10, 50 and 100 cells/mL. Across a final range of spiked cell concentrations between 9–129 cells/mL, the Parsortix platform demonstrated a mean cell capture rate of 53.5% (95% CI: 50.394–56.696) (Figure 2A). There was no significant correlation between spiked cell concentration and capture rate across both cell lines (Spearman’s r = 0.365, *p* = 0.221) and no observed trend within individual cell lines. Dividing the data into three groups of target cell concentrations of ≈10, 50 and 100 cells/mL blood, the mean capture rate was marginally lower for 10 cells/mL compared to 50 and 100 cells/mL, although not to a significant level (ANOVA. *p* = 0.415).

An alternative method of CTC enrichment uses negative depletion of CD45-positive cells via MACS; we compared this method to Parsortix microfluidic enrichment. When evaluating capture rates of a target cell count of 100 cells/mL whole blood, Parsortix demonstrated significantly increased cell capture (57.0% vs. 28.0%, respectively, *t*-test, *p* = 0.0008) (Figure 2C).

### 3.2. Cell Preservation and Sample Storage Time

**Figure 3:**
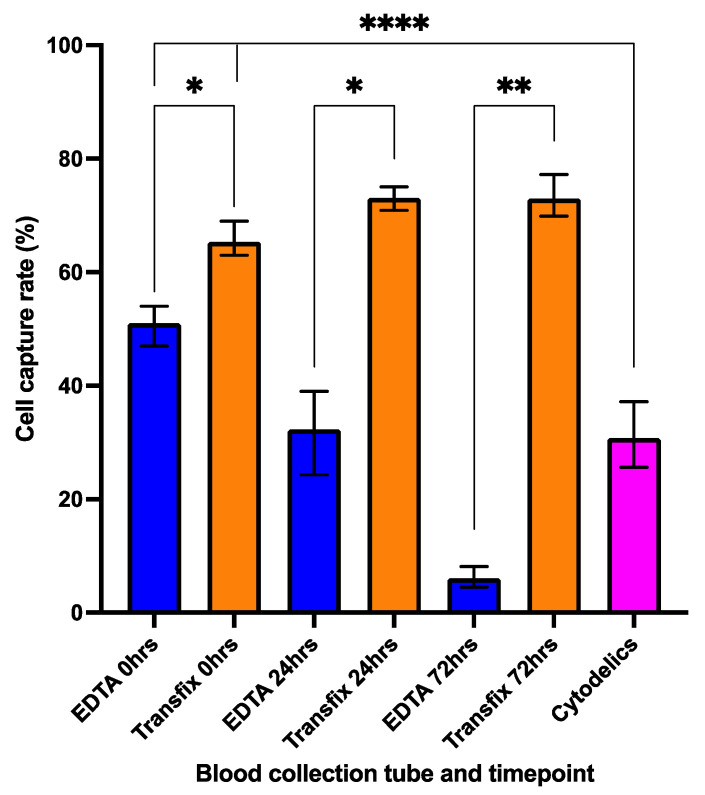
Improved cell capture rate with Transfix compared to EDTA and Cytodelics. Healthy donor (HD) blood (9 mL) collected into EDTA or Transfix Blood collection tubes and spiked with 100 cells/mL (target concentration), then enriched using Parsortix at timepoints 0 h, 24 h or 72 h. 1 mL of HD blood spiked with 100 cells and preserved using Cytodelics protocol and frozen to −80 °C prior to thawing and processing. Bar denotes mean cell capture rate and whiskers range of values for *n* = 3 (EDTA, Transfix) or *n* = 5 (Cytodelics experiments). (* = *p* < 0.05, ** = *p* < 0.001, **** = *p* < 0.0001).

Having established a baseline Parsortix capture rate for HNSCC cells, we compared BCTs at different timepoints. At baseline 0 h (sample processed immediately but requiring ~4 h processing time) Transfix-collected samples demonstrated a significantly higher capture rate of 65.3% compared to 51.0% for EDTA samples (*p* = 0.0329). At subsequent timepoints (24 h and 72 h) the capture rate of Transfix preserved samples was stable (73% and 73.1%, respectively) and significantly higher than the corresponding EDTA sample capture rate at each timepoint (*p* = 0.0145 and 0.0018, respectively). Across all timepoints, compared to baseline levels, the drop in EDTA cell capture rate was significant, decreasing to 32.3% at 24 h (*p* = 0.0236) and 6.0% at 72 h (*p* = 0.0016). The Cytodelics kit demonstrated a mean capture rate of 30.7%, which was significantly less than both EDTA and Transfix baseline 0 h values, but comparable to the EDTA capture rate at 24 h (Figure 3).

### 3.3. Impact of Parsortix Enrichment on Gene Expression Patterns of Unfixed HNSCC Cell Lines

**Figure 4:**
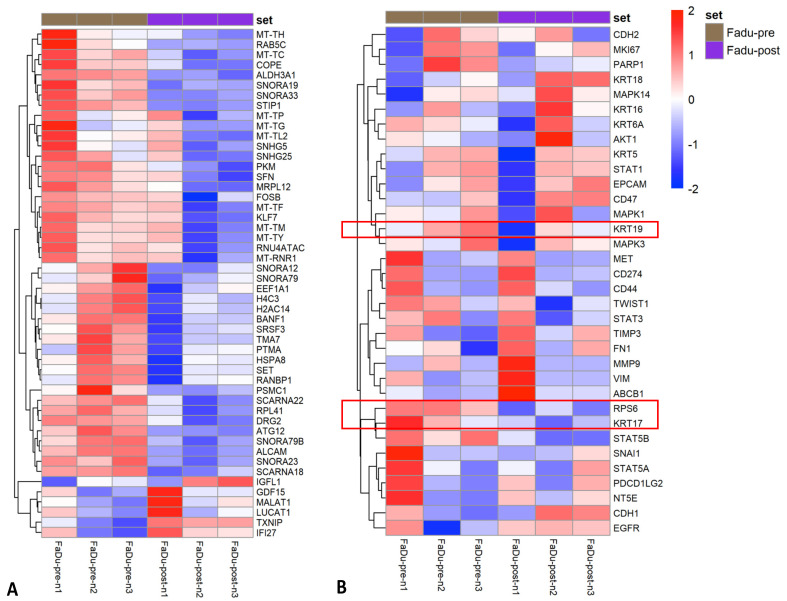
Differential gene expression analysis of FaDu cell line before and after Parsortix microfluidic enrichment demonstrates a cell stress response and downregulation of keratin proteins. Heatmap of differential gene expression analysis of paired unfixed FaDu cells before and after Parsortix enrichment. (**A**) The top 50 differentially expressed genes are displayed, the majority of which are downregulated and involved in mRNA/RNA and ribosomal protein processing. Upregulated genes indicate an oxidative stress response. (**B**) Heatmap of gene expression for specific HNSCC/CTC target markers. Three genes (keratins 17 and 19 and ribosomal protein S6, highlighted within red boxes) demonstrated differential expression and downregulation after Parsortix enrichment; the remaining genes were not differentially expressed before and after enrichment.

Cell fixation with formaldehyde halts cellular metabolic activity via protein cross-linking. The stabilisation and “freezing” of CTC metabolic activity at the time of sample collection using a fixation BCT, such as Transfix, provides the benefit of protecting fragile CTCs from changes in gene and/or protein expression during potentially traumatic enrichment protocols. However, the majority of CTC research to date has used unfixed blood samples. To assess transcriptional changes occurring during microfluidic enrichment, the gene expression profile of unfixed FaDu cells (*n* = 3 paired samples) was assessed pre- and post-processing. Differential gene expression (DGE) analysis revealed downregulation of several genes in Parsortix-enriched cells (Figure 4A). Gene ontology enrichment analysis revealed significant downregulation of gene sets involved in mRNA and RNA processing, RNA binding and ribosome/protein processing and binding (Table 2). Decreased transcriptional function is a sign of cells moving from a rapidly dividing phenotype into a quiescent phase, a potential response to cell stress [26]. No significant GO gene set upregulation was observed. However, at the single gene level, genes related to cell injury (*GDF15* and *ZNF557*), apoptosis (*MALAT1*, *TXNIP* and *TRIML2* [27]) and oxidative stress (*MMP1*, *ZNF721*, *HILPDA*, *SOD2*, *MAP2K3* and *WSB1*) were upregulated in the enriched cell group. We compiled a list of 34 markers associated with HNSCC [28] and CTC identification [29], including druggable targets, key pathway proteins and markers of epithelial-mesenchymal transition (EMT), stemness and proliferation/metastasis. Of these genes, 3 had been shown as differentially expressed in the above analysis—keratins 17 and 19 and ribosomal protein S6 were downregulated. Expression of EMT and stemness markers, and druggable targets were not significantly altered (i.e. differentially expressed) by the enrichment process (Figure 4B).

### 3.4. RNA Extraction and Sequencing of Transfix Preserved Cells

To allow gene expression analysis of CTCs collected in Transfix BCTs and enriched using Parsortix, we trialled various RNA extraction kits. While the contents of Transfix BCTs is proprietary knowledge, it is assumed to be some form of fixative agent, likely formaldehyde based. Formaldehyde fixation creates cross-linkages between proteins, between nucleic acids, and between proteins and nucleic acids making RNA extraction and subsequent sequencing very challenging. Several RNA extraction kits are optimised for formalin fixed paraffin embedded (FFPE) tissues. However, currently none are specifically designed for fixed and low input samples, as encountered after Parsortix enrichment of Transfix BCT samples. We tested several extraction kits on concentrations of 100, 1000, 10,000 and 100,000 FaDu cells incubated in Transfix preservative for 24 h. The ThermoFisher PicoPure RNA isolation kit was able to successfully extract RNA (RNA integrity number (RIN) > 7) from Transfix preserved cells down to an input of 1000 cells. Extracting RNA using the PicoPure kit from Parsortix enriched cells (from samples of cell line spiked Transfix blood) demonstrated a mean RIN value of 7.2 (*n* = 3, range 6.7–7.7). This is comparable to a RIN value of 8.7 (*n* = 3, range 8.4–9.2) when performing the same experiment on non-fixed (EDTA BCT) Parsortix enriched cells.

**Figure 5:**
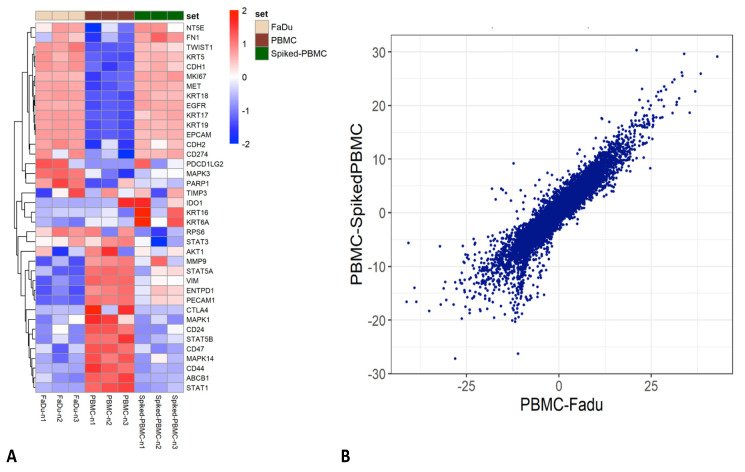
Proof-of-principle that a rare cell population (epithelial CTC) can be identified using bulk sequencing of Transfix preserved, Parsortix enriched blood samples. Analysis of gene expression from “bulk sequencing” of Transfix preserved and Parsortix enriched healthy donor blood samples spiked with 1000 FaDu cells (*n* = 3). (**A**) Heatmap to visualise gene expression of the aforementioned HNSCC/CTC target gene list. Note that epithelial and key HNSCC pathway genes are highly expressed in the “spiked-PBMC” population, indicating the presence of enriched epithelial (FaDu) cells. (**B**) Scatterplot of differentially expressed genes between the Parsortix enriched “spiked-PBMC” samples and PBMC controls vs. the FaDu and PBMC control samples. Axes denote Z-score as a measure of number of standard deviations of the significant difference in gene expression between samples, i.e., negative values are downregulated genes and positive values upregulated genes. High correlation (Pearson R = 0.933, Spearman R = 0.934, *p* < 0.001) confirms the presence of FaDu specific, i.e., rare epithelial cell, transcripts detectable from the enriched rare cell population.

Following this, we sought to confirm that gene expression analysis (via mRNA transcript counting) was possible on Transfix-preserved and Parsortix-enriched cells, and to demonstrate proof-of-principle that transcripts from a rare CTC (epithelial cell) population could be identified when performing “bulk” sequencing of the Parsortix-enriched cell fraction. FaDu cell spiked HD blood samples were enriched using Parsortix (time-to-processing 24 h) and mRNA transcript counting performed. Matched HD PBMCs were used as a negative control and FaDu cells as a positive control. DGE analysis between the Parsortix-enriched cell fraction and the PBMC controls revealed the presence of numerous epithelial cell specific transcripts-including EpCAM, e-cadherin (CDH1), and cytokeratins (KRT) 5, 17, 18 and 19 (Figure 5A). These markers—routinely used for CTC identification in HNSCC—were highly expressed in the FaDu positive-control cells. As an analytical exercise to confirm the presence of FaDu cells in the enriched samples, DGE of the Parsortix-enriched samples and PBMC controls was correlated to DGE of the PBMC and FaDu control samples. A high correlation was observed between the two datasets (Pearson R = 0.933, Spearman R = 0.934, *p* < 0.001, Figure 5B) indicating that HNSCC-specific gene expression might be successfully identified from CTCs using Transfix BCTs and Parsortix enrichment.

### 3.5. Enrichment of Transfix Blood Samples from HNSCC Patients

**Figure 6:**
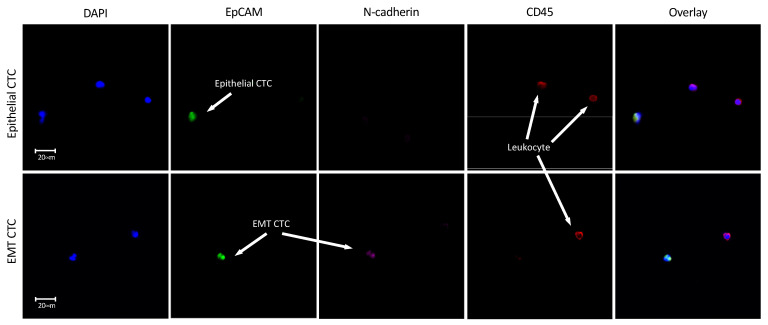
Immunofluorescent images from patient no. 1 demonstrating different CTC phenotypes. Images obtained on a Leica DM6000B microscope at 40× magnification. Top row-an EpCAM+ CD45− (DAPI+) epithelial CTC is labelled next to two EpCAM- CD45+ leukocytes. Bottom row–a CD45- EpCAM+ cell also with low expression of N-cadherin, indicating the process of a transition from an epithelial to mesenchymal (EMT) phenotype.

Having established the optimal protocol for HNSCC cell isolation in a model system, we next sought to identify CTCs in Transfix blood samples taken from HNSCC patients as proof-of-principle for future research. We evaluated a pilot cohort of four patients with advanced stage III/IV HNSCC, who were specifically chosen to give the highest probability of CTCs being present and thus validate our protocol. Two of these (patients 1 and 3) had nodal disease (stage N2b and N1 respectively) while the other two patients did not. Cells meeting criteria for CTCs (DAPI positive, CD45 negative and EpCAM and/or N-cadherin positive) were identified in the the blood of two patients (Table 3). CTCs expressed various combinations of phenotypic markers, indicating mixed sub-populations including EpCAM positive epithelial CTCs and epithelial-mesenchymal transitioning CTCs with expression of both EpCAM and N-cadherin (Figure 6). The two patients who had EpCAM+ CTCs both had nodal disease and their epithelial CTCs were accompanied by mesenchymal transitioning CTCs.

## 4. Discussion

The Parsortix microfluidic platform is now optimised to detect CTCs from multiple cancer types, including lung [30], breast [31], and prostate [32], among others. Crucially, these studies have provided proof-of-concept that molecular and genomic characterisation of CTC populations is possible following microfluidic enrichment. Our findings demonstrate evidence for the utility of the Parsortix platform to enrich CTCs in HNSCC-building on previous reports and providing the first use of fixation/preservative BCTs to improve CTC capture rates in HNSCC. Our cell line optimisation model demonstrated a mean capture rate of 54% for unfixed cells, which is comparable to similar optimisation reports for other cancer cell lines. Hvichia et al. evaluated Parsortix enrichment of five cancer cell lines (pancreatic, melanoma, prostate, lung and bladder) spiked into whole blood at concentrations of 10, 50 and 100 cells/mL [16]. Capture rates across the different cell lines ranged from 42–70% and demonstrated an inverse correlation with cell size. The smaller T24 bladder cancer cell line (mean cell size 18 μm) had the lowest capture rate of 42%. It is interesting to note that despite a smaller mean cell size of 12.2 μm, our capture rate for the two HNSCC cell lines was relatively high, potentially reflecting the differing bio-physical properties of cells from various cancers. Several microfluidic platforms have been described to enrich CTCs from whole blood; however, it should be noted that size-based CTC enrichment may be adversely impacted by heterogeneity of CTC morphology [6,33,34]. While it is outside the scope of this article to discuss all of the results obtained across the various CTC enrichment platforms, our results of capture rates for spiked in cell lines appear comparable. Clearly no CTC enrichment platform, be it size or marker dependent, is perfect and choice may depend upon the required downstream analysis. In a direct comparison to the Epcam-dependent CellSearch^®^ platfom, Parsortix was shown to be superior in HNSCC at detecting CTCs with heterogenous EMT marker expression [35]. Of note is the difference that cancer type and cell line may have upon capture rates and hence the difficulty in direct comparison of datasets. In a HNSCC cell line model, Kulasinghe et al. have reported results of “spiral” and “straight” chip microfluidic platforms, similar to other devices [9,36]. They demonstrated capture rates of 60–70% and 40–80%, respectively for these two chip designs, down to 10 cells/mL blood, comparable with our results in fixed cells.

The method of cell preservation and time to processing is an important consideration in CTC research, given reports of the fragility of cells and a half-life of ≈ 30 min [37]. Numerous blood collection tubes (BCTs) have been utilised in research to-date. These can be divided into those that are not specifically designed for CTC enrichment and that contain an anticoagulant, such as EDTA or Citrate BCTs, or CTC/cell-free DNA-specific BCTs, for example, the cell-free DNA Streck^®^ BCT, Transfix^®^ CTC BCT (Cytomark) or CellSave^®^ (Cellsearch). Such variety in current practice is partly driven by the fact that the output metric of CTC research may govern the method of CTC collection and stabilisation. In cases when enumeration of CTCs is the primary goal, then cell membrane stabilisation is the priority to preserve whole viable cells, particularly when samples are not processed immediately. However, RNA/DNA extraction from preservative-containing BCTs can be difficult. Several studies have reported increased recovery rates of spiked cells using DNA-preservative BCTs (Streck) when compared to EDTA BCTs <24 h processing [13,14]. Yet, at 72 h, their benefit appears to drop and cell capture rates decrease [14], indicating that CTC-specific preservative BCTs may be superior in samples stored beyond this timepoint. To date, only one study has assessed Parsortix CTC enrichment in combination with a CTC preserving BCT. Using a breast cancer cell line model, Koch et al. compared capture rates of Parsortix enriched spiked blood samples in EDTA BCTs at 0 h and CellSave, Streck and Transfix BCTs at a 24 h time point only [15]. The Transfix BCT was comparable to EDTA collected samples (64% vs. 60.7%), but far superior to the CellSave and Streck preservative BCTs (16.7% and 22.7%, respectively). However, this group did not evaluate EDTA and Transfix BCTs at equal timepoints (i.e., EDTA at 24 h) or storage time greater than 24 h. Our data demonstrate that across all timepoints Transfix BCTs returned a significantly higher cell capture rate than EDTA BCTs, with a marked difference at 72 h (73% vs. 6%, *p* = 0.0016). We argue such data are of crucial importance if samples are being collected as part of multi-centre clinical trials and time to processing is increased due to sample transport. Thus, variation in time between sampling and processing of unfixed samples has the potential to introduce significant bias if CTC enumeration is used as a biomarker. Furthermore, it is unknown if CTC phenotypic subsets (i.e., epithelial vs. mesenchymal) have differential sensitivities to cell storage, which would further impact data reliability for unfixed stored samples.

RNA degradation within CTCs has been shown to occur within a matter of hours [38]. The effect of the enrichment technique on gene expression of CTCs is unclear. Physiological stress will alter the gene expression of cells, and consequently what is thought to be a snapshot of a CTC expression profile may actually be a biased result due to the sample handling protocol. However, while multiple studies have evaluated RNA yield and gene expression of CTCs at various processing timepoints, few have directly compared gene expression before and after CTC enrichment. Powell et al. assessed the effect of an immunomagnetic CTC enrichment protocol (Mag Sweeper) on the expression of 15 target genes in a breast cancer cell line model [39]. No variation in gene expression was demonstrated before and after enrichment. To the best of our knowledge, our study is the first to present gene expression data before and after microfluidic enrichment. Our findings indicate that unfixed cells (i.e., those collected in an EDTA BCT) undergo trauma which elicits a stress response with evidence of oxidative stress and early apoptosis. Cytokeratins have a key role in epithelial cell protection from mechanical and also non-mechanical stress (such as hypoxia), and in such conditions undergo reorganisation and upregulation or downregulation of specific keratin proteins [40]. We report that keratins 17 and 19 were downregulated in unfixed microfluidic enriched epithelial cells. The clinical significance of such data could be profound, particularly if specific cytokeratins are being targeted for CTC identification or prognostic stratification in specific cancer types. However, cytokeratins are abundant proteins in epithelial cells with high baseline expression, therefore a decrease in expression of certain cytokeratins (even if significant) may be acceptable for detection purposes when using antibodies targeting multiple cytokeratin proteins. Furthermore, our cell line samples were compared at baseline (0 h time to processing) and it is unknown whether prolonged storage or processing of unfixed samples could cause further keratin dysregulation—a variable which should be internally validated in other enrichment protocols, or consideration made to use preservative BCTs such as Transfix. Aside from keratin genes, expression of EMT and druggable target genes were not significantly altered in the enriched cell line. It is acknowledged that functional protein expression may differ or be impacted separately by CTC enrichment. Multiparameter proteomic assessment would be required for this evaluation.

Several reports have detailed complex lab-based protocols to stabilise CTCs prior to microfluidic enrichment [38]. We favour utilising a commercially available BCT that fixes CTCs at the point of collection—facilitating sample collection by staff in a busy hospital environment, eliminating the need for staff time, specialised equipment or staff training on sample pre-processing and enabling transport between sites in multi-site clinical trials—thus, preserving the CTC phenotype and allowing unbiased data analysis. While previous studies have raised concerns regarding RNA sequencing on fixed/preserved CTCs [41], we have demonstrated how gene expression profiling is possible using Transfix samples. Epithelial cell transcripts from a Parsortix enriched population could be identified in Transfix preserved blood samples to confirm the presence of rare CTCs-using a “bulk” sequencing method as described in previous studies [42,43]. Excluding initial equipment outlay and staffing costs (which will differ between institutions and countries), the cost to analyse one sample in 2021 is estimated to be in the region of 110 GBP/150 USD/130 EUR.

Our findings from patient samples demonstrate that Transfix BCTs together with Parsortix enrichment can be used to successfully identify CTCs in HNSCC. The initiation of an EMT is seen as a key step in the pathway of cancer cells acquiring a malignant phenotype, leading to metastasis [44]. Therefore, it is crucial to be able to identify and quantify these CTCs in HNSCC as they may serve as prognostic biomarkers and/or targets for therapeutic intervention. The addition of a mesenchymal marker to the accepted epithelial marker-positive CTC identification criteria allowed us to define sub-populations of CTCs undergoing or potentially having undergone an EMT and demonstrating low epithelial marker expression [7,8]. Such data demonstrate the necessity of a marker-independent protocol, such as Parsortix, to capture cells having undergone a partial or full EMT that are epithelial marker low (or negative). Although in a small cohort, the observation of epithelial and EMT CTCs correlating with nodal disease is noteworthy and potentially a glimpse of the phenotypic switch occurring to enable tumour progression and regional and/or distant metastasis. We accept that such a small cohort is a limitation of the data presented; however, it is presented as early proof-of-concept data only. A larger cohort study is currently being conducted. Ultimately, to fully elucidate the molecular and phenotypic heterogeneity of proposed CTC sub-populations, multi-omic single-cell characterisation is required-currently not achieved in HNSCC, but reported in other cancer types [45].

## 5. Conclusions

We present primary evidence highlighting the benefit of using the fixation Transfix BCT when processing samples 24–72 h after collection using Parsortix enrichment; allowing ease of collection, storage and transport, particularly useful in multi-site clinical trials. Microfluidic enrichment of un-fixed cells induces a cell stress response related to oxidative stress, with downregulation of certain cytokeratin genes and the ribosomal protein S6 gene. We strongly argue that the next stage in validating CTC research-moving towards large cohort clinical trials, particularly in HNSCC-requires the development of a standardised protocol for CTC detection, enumeration and/or downstream multi-omic characterisation. Our findings serve as a foundation to use Transfix BCTs with the Parsortix platform to achieve this goal and identify potential prognostic and/or predictive biomarkers for clinical translation.

## Figures and Tables

**Table 1:** Antibody panel optimised for detection of CTCs enriched using Parsortix having been collected in Transfix blood collection tubes (PE—phycoerythrin, APC—Allophycocyanin).

Cell Phenotype Marker	Antibody	Host Species	Type	Clone	Flourochrome	Source
epithelial	EpCAM (CD326)	mouse	Monoclonal, conjugated	9C4	Alexa Fluor 488	Biolegend
mesenchymal	N-cadherin (CD325)	mouse	Monoclonal, conjugated	8C11	APC	Biolegend
pan-leukocyte	CD45	mouse	Monoclonal, conjugated	2D1	PE	Biolegend
nuclear dye	DAPI					

**Table 2:** Downregulated differentially expressed genes in Parsortix enriched FaDu cells demonstrate enrichment of mRNA/RNA and ribosomal processing gene sets. Functional enrichment analysis of gene ontology (GO) gene sets (biological process, cell component and molecular function) of downregulated genes in the Parsortix processed FaDu cells. Top five for each domain are displayed. There is a clear pattern of downregulation in mRNA/RNA processing and ribosomal proteins as seen in cells undergoing a stress response and/or apoptosis.

Gene Ontology (GO) Category and Gene Set	Gene Set Size	Direction	*p* Value	q Value
**GO Biological process**				
GO:0016071 mRNA metabolic process	750	Down	1.30 × 10^−7^	0.000694
GO:0006402 mRNA catabolic process	330	Down	4.70 × 10^−7^	0.000975
GO:0006396 RNA processing	884	Down	6.20 × 10^−7^	0.000975
GO:0006412 translation	560	Down	7.33 × 10^−7^	0.000975
GO:0022613 ribonucleoprotein complex biogenesis	423	Down	1.09 × 10^−6^	0.000999
**GO Cell component**				
GO:1990904 ribonucleoprotein complex	637	Down	2.79 × 10^−10^	0.000000194
GO:0005730 nucleolus	843	Down	0.000000324	0.000113
GO:0005840 ribosome	210	Down	0.000000945	0.000219
GO:0044391 ribosomal subunit	174	Down	0.00000153	0.000266
GO:0022626 cytosolic ribosome	99	Down	0.00000884	0.00123
**GO Molecular function**				
GO:0003723 RNA binding	1452	Down	2.20 × 10^−14^	1.98 × 10^−11^
GO:0003735 structural constituent of ribosome	151	Down	1.91 × 10^−6^	0.000779
GO:0044877 protein-containing complex binding	836	Down	2.60 × 10^−6^	0.000779
GO:0005198 structural molecule activity	374	Down	7.24 × 10^−6^	0.00162
GO:0045296 cadherin binding	291	Down	9.66 × 10^−5^	0.0174

**Table 3:** Patient clinicopathological data and presence of CTCs. Cells that were DAPI-positive and CD45-negative with expression of CTC markers (EpCAM and/or N-cadherin) were identified in both patients 1 and 3, both of whom had advanced stage III/IV HNSCC with node positive disease. For both of these patients epithelial CTCs and epithelial-mesenchymal transitioning CTCs were detected.

Pt. ID	Age/Sex	Site	Stage	EpCAM+ CTC (DAPI+, CD45−)	EpCAM+/N-cadherin+ CTC (DAPI+, CD45−)
**1**	84 F	Oral	T4a N2b M0	+	+
**2**	74 M	Laryngeal (recurrence)	T4a N0 M0	−	−
**3**	69 F	Oral	T4a N1 M0	+	+
**4**	73 M	Hypopharyngeal	T3 N0 M0	−	−

## Data Availability

The data presented in this study are available upon request from the corresponding author.
